# Novel applications of linked administrative data – adding longitudinal capability and additional variables to a nationally representative survey of an indigenous population.

**DOI:** 10.23889/ijpds.v9i5.2817

**Published:** 2024-09-18

**Authors:** Tori Diamond, Matt Edwards, Andrew Sporle

**Affiliations:** 1 The University of Auckland; 2 iNZight Analytics

Can linked administrative data be used to transform New Zealand's only sample survey on indigenous wellbeing into a longitudinal study?

This project extends the usefulness of an important survey dataset by linkage to admin data, effectively adding longitudinal capability within a linked administrative data source. This created robust statistical processes to transform an official statistics survey into a nationally representative cohort study.

NZ's Integrated Data Infrastructure (IDI) is a research database of administrative and survey datasets containing a range of variables linkable at the individual level. Te Kupenga is a large nationally representative post-censal survey of NZ's indigenous population (Māori) and is the only official survey with Māori culturally-informed variables. However, it is under-utilised in research.

The Te Kupenga survey was used as a foundational cohort linking to outcomes and determinants in different datasets at different time periods. Outcomes included hospitalisations and COVID-19 vaccinations, while determinants included individual, household and geographic variables.

Linking a representative survey to admin data created issues of loss to follow-up and missing data, so the original sample is not maintained after linkage. Loss to follow-up and missingness differed depending on variable selection and time periods. So, new universally applicable weights were not possible. However, we created a robust, generally applicable process for re-weighting survey data to account for missingness and loss to follow-up in admin data.

This project demonstrates the approach for turning a sample survey into a longitudinal cohort using admin data and creates methods that can be used for other official statistics surveys.

